# Antibiotics and Antimicrobials Resistance: Mechanisms and New Strategies to Fight Resistant Bacteria

**DOI:** 10.3390/antibiotics11030400

**Published:** 2022-03-17

**Authors:** Cécile Muller

**Affiliations:** UNICAEN, Research Unit of Microbial Risks U2RM, Normandie University, 14032 Caen, France; cecile.muller@unicaen.fr

Antibiotics have revolutionized medicine over the past century and have helped to control most infectious bacterial diseases. However, misuse, overuse, and agricultural use of these antibiotics have led to the emergence of resistance phenomena, which are now considered by the WHO to be one of the most serious threats to global health, food security, and human development [1]. Moreover, public information is needed to help reduce antibiotic resistance: appropriate treatment, curative or prophylaxis, combinations or not, duration, and all these parameters should be understood by patients to enable them to be aware of long-term risks, which is necessary to help manage the crisis.

Considering the increase in antimicrobial resistance (AMR), a growing awareness on the search for new antibacterial agents is essential. Nevertheless, programs for the discovery of new antibiotics may take more than 15 years of research [2], even though recent “machine learning” approaches have been implemented [3]. The pipeline conventionally used is to structurally modify molecules known to circumvent resistance problems, and to increase pharmacokinetics, activity spectra or safety. However, multiple mechanisms of cross-resistance remain [4], and, in the long term, it will be necessary to put in place the innovations necessary to find drugs directed against new targets to avoid resistance. Other active compounds, such as antimicrobial peptides, which are produced by numerous life form are interesting leads, but are also exceptions, such as gramicidin (the only one to be validated by the Food Drug Administration for human application) and teixobactin isolated from the non-cultivable bacterium *Eleftheria terrae* [5].

Consequently, new treatment options are urgently needed to get out of this gridlock, like complementary therapies allowing to increase the effect of antibiotics, targeting virulence or regulation of resistance genes expression, like quorum-sensing. Thus, a new strategy has emerged recently: instead of killing bacterial pathogens, the concept is to disarm them by targeting and blocking virulence mechanisms. Antivirulence drug can be used as supplements to traditional antibiotics [6,7], making bacteria less likely to become resistant to combination drug therapy, and are consequently expected to reduce antibiotic use. Antiviral molecules, which in some cases do not affect bacterial growth and thus limit the risk of generating resistance, can be used as adjuvants to antibiotics to increase their therapeutic effects. Thus, certain compounds can also have an impact on the biofilms formation, often associated with treatment failures, or on systems regulating the production of virulence. It is therefore important to study the possible potentiating effects of active compounds with antibiotics.

Three contributions in this Special Issue focus on antibiotic resistance profile of strains isolated in hospitals [8,9,10]. The study by Udo et al. [8] evaluated the prevalence of chloramphenicol and methicillin resistant *Staphylococcus aureus* clones, due to the presence of the FexA exporter, allowing the resistance to an analog of chloramphenicol (florfenicol) in Kuwait hospitals. These variants were found to probably arise from strains isolated from chicken meat, and the presence of virulence factors was also evaluated. The contribution of Bandy and Tantry was focused on Enterobacteriaceae and their resistance profile and extended-spectrum beta-lactamase activity in Saudi Arabia [9]. Seasonal variations were observed, as well as high tigecycline resistance, with important repercussions on patient treatments. A genomic approach was used by Borelli et al. to investigate antibiotic resistance gene prevalence and the association of these genes with mobile genetic elements, in several species isolated within Brazilian hospital [10]. This study illustrates the interesting progress that whole-genome sequencing can offer to follow dissemination of antibiotic resistance among clinical strains, and to anticipate on multidrug resistant bacteria outbreaks.

The work of Teng et al. [11] summarized data on reported infections due to *Elizabethkingia* sp., an emerging nosocomial pathogen with an antibioresistance profile. This study also described on a *E. anophelis* isolate of an intra-abdominal infection, and used a genomic approach to investigate antibiotic resistance genes.

The last two contributions of this Issue are reviews focusing on new strategies to fight antibioresistant bacteria. Baëtz and co-workers [12] described the current understanding of the treatment used against *Staphylococcus* and *Enterococcus* vancomycin-resistant bacteria, like antibiotic treatments (conventional or modified) or non-traditional antimicrobials. This last part concerns antimicrobial peptides, bacteriocins, bacteriophages or nanoparticles: all these advances could contribute to reduce the use of antibiotics, and thus lower the selection pressure for multi-resistant bacteria.

The review by Raza et al. [13] discussed the capacity of bacteria to acquire resistance to antibacterial agents excluding antibiotics. Physical factors, nanomaterials and bacteriophages are promising approaches to tackle multidrug resistant bacteria, but these microorganisms can also develop mechanisms to protect themselves from these antimicrobials, such as CRISPR against bacteriophages infections.

The main message of contributors is that we are defenseless in the face of multidrug resistant bacteria, that was described as a major global health problem. Solutions to fight AMR do exist, such as identifying strategies that can work to reduce the burden of bacterial antibiotic resistance. Combating AMR will also require collective action, and multisectoral collaboration and partnerships between all actors worldwide is necessary, like governmental and non-governmental agencies, pharmaceutical companies, researchers, public health practitioners, hospital administrations, patients, and agriculture industry leaders [14].
